# Contrast-Enhancing Snapshot Narrow-Band Imaging Method for Real-Time Computer-Aided Cervical Cancer Screening

**DOI:** 10.1007/s10278-019-00215-1

**Published:** 2019-05-08

**Authors:** Dingrong Yi, Linghua Kong, Yanli Zhao

**Affiliations:** 1 College of Mechanical Engineering and Automation, Huaqiao University, Xiamen, 361021 China; 2 School of Mechanical and Automative Engineering, Fujian University of Technology, Fuzhou, 350118 China

**Keywords:** Contrast enhancement, Cervical cancer screening, Snapshot narrow-band imaging, Computer-aided diagnosis, Euclidean distance algorithm, Optical biopsy

## Abstract

Composition of cervical precancerous lesions and carcinoma in situ is rich in hemoglobin, unlike healthy tissues. In this study, we aimed to utilize this difference to enhance the contrast between healthy and diseased tissues via snapshot narrow-band imaging (SNBI). Four narrow-band images centered at wavelengths of characteristic absorption/reflection peaks of hemoglobin were captured with zero-time delay in between by a custom-designed SNBI video camera. Then these spectral images were fused in real time into a single combined image to enhance the contrast between normal and abnormal tissues. Finally, a Euclidean distance algorithm was employed to classify the tissue into clinical meaningful tissue types. Two pre-clinical experiments were conducted to validate the proposed method. Experimental results indicate that contrast between different grades of diseased tissues in the SNBI generated image was indeed enhanced, as compared to conventional white light image (WLI). The computer-aided classification accuracy was 100% and 50% as compared to the gold standard histopathological diagnosis results with the SNBI and the conventional WLI methods, respectively. Further, the boundary contour between health tissue, cervical precancerous regions, and carcinoma in situ can be automatically delineated in SNBI. The proposed SNBI method was also fast, and it generated automatic diagnostic results with clear boundary contours at over 11 fps on a Pentium 1.6-GHz laptop. Hence, the proposed SNBI is of great significance to enlarge worldwide the coverage of regular cervical screening program, and to live guide surgeries such as biopsy sample collection and accurate cervical cancer treatment.

## Background

Among various imaging methods including magnetic resonance imaging, ultrasound, and computerized topography, contrast improvement is the ultimate goal for all medical imaging. In terms of the widely used medical white light imaging (WLI) (e.g., various cavity mirror and endoscopic), although it provides a higher resolution and faster imaging speed than MRI and CT to help medical doctors to discern changes of finer lesions and to better reduce or eliminate the blurring effect caused by heart bumping or pulmonary motion of the patient, however, its contrast is quite limited. With the conventional WLI, various complicated techniques including spatial registration, segmentation, lesion detection, and classification were required to process the image and differentiate the tissue into different clinical meaningful types [1]. For example, during the global war to fight against the cervical carcinoma, which is the second most common gynecological malignancy in the world [2], it is found that with the contrast provided by WLI, it is hard to build an accurate computer-aided diagnosis method to automate the cervical cancer screening process. While a CAD cervical cancer screening method is expected to improve the coverage of regular cervical cancer screening program and greatly prevent the occurrence of cervical cancer in underdeveloped countries and low-income regions [2]. Accordingly, considerable efforts have been dedicated in the last two decades to automate the analysis of white light colposcopic images to support the medical decision process and to provide a data-driven channel for communications of findings [1]. Recently, Intel and Mobile ODT organized a competition for the automatic analysis of digital colposcopies [1]. Fernandes and colleagues gave a comprehensive review and summary of existing computer-aided diagnostic (CAD) method applicable to images provided by a conventional white light digital colonoscopy. They concluded that the existing WLI methodologies require a significant amount of manual labeling, including spatial localization of the lesions at an image level [1]. To deepen the degree of automation of the cervical cancer screening process or any other lesion diagnosis process involving optical imaging, one important direction of effort is to enhance the contrast at the image acquisition stage, i.e., at the signal acquiring stage instead of at only the signal processing stage.

Accordingly, multiple authors suggest that spectral imaging method which combines the advantages of both digital image and spectral analysis may enhance the contrast between normal and abnormal tissues [3, 4]. Spectral imaging may simplify the CAD process and reduce the amount of expert manual labeling. The fundamental principle of spectral imaging is that different chemical and biological molecules have a different, unique, and wavelength-dependent way to reflect, scatter, or absorb light. Accordingly, it is possible to use spectral difference to differentiate diseased regions, or to make optical biopsy. With the spectral difference-enhanced optical biopsy, a CAD method can be established for automatic and objective diagnosis of the precancerous lesions. Sterenborg proposed a spectral imaging method for diagnosing cervical tissues using an optical fiber to collect reflective lights from cervical squamous and achieved 89 and 80% of sensitivity and specificity, respectively [5]. Recently Wang and coworkers proposed a multi-scale hyperspectral imaging method to detect cervical neoplasia at both tissue and cellular levels [6]. The advantages of various existing spectral imaging based optical biopsy methods are non-invasive, objective, and has the potential to reduce the number of unnecessary biopsies. However, existing spectral imaging method obtains data by sequential scanning in the spatial domain to cover the area to be diagnosis. Therefore, it is time consuming to acquire the diagnosis needed data—it takes a while for existing spectral or hyperspectral imaging methods to scan the whole area of the cervix. Other limitations include the need for spatial registration, non-uniform illumination, high-cost and bulky setup, and sophisticated image processing [7]. Instead of using spectral or hyperspectral imaging, it was found that as few as only three specific spectral bands are sufficient to classify the cervical tissues into normal, inflammation, and high-grade lesions [7]. This is consistent with Benavides’s report that reflectance MSI at only a couple of wavelengths is needed to differentiate cervical cancer from its background [8]. Multispectral reflectance imaging is highly preferable over hyperspectral imaging as it is more efficient, more compact, and cost effective [7]. This is because unlike spectral or hyperspectral imaging, MSI does not spend time or effort to collect images of unwanted spectral bands. To further explore MSI, some researchers combine reflectance MSI with autofluorescence to investigate the combined value of both. Ren and coworkers proposed to combine multispectral reflectance, autofluorescence, and RGB imaging for noninvasive characterization of cervical intraepithelial neoplasia (CIN) [9]. Their preliminary clinical results indicate that the added value of autofluorescence to the reflectance MSI is arguable considering the economic cost of the device and the additional operation in a clinical setting. So far, there is a trend to capture a couple of reflective spectral images to enhance the visual contrast in order to help the lesion-associated characteristic features stand out. For example, Olympus’s narrow-band imaging (NBI) method uses only two narrow bands corresponding to absorption characteristics of hemoglobin to enhance contrast to ensure that blood vessels in the mucosa stand out clearly [10]. NBI method is successfully used for diagnosing bladder cancer [11, 12], early gastric cancer [13], pulmonary diseases [14, 15], colon cancer [16, 17], and cervical adenocarcinoma [3, 11]. However, in most existing reflectance MSI including the Olympus NBI, the spectral images are sequentially acquired. They all involve scanning in the temporal domain. Hence, the spatial dislocation between images is inevitable, and spatial registration algorithm is required, which increases the computational load and reduces the efficiency.

Driven by the direct clinical needs of enhancing visual contrast for detecting early staged cancers at their early stages with a low-cost, competent, objective, convenient method, in this paper, we demonstrate a semi-automatic miniaturized snapshot narrow-band imaging (SNBI) method. Although the objective of SNBI is to enhance image contrast and to provide objective diagnosis, in this paper, it is evaluated for the tasks of cervical cancer screening. This is because it is of crucial importance to have a fast and automatic cervical cancer screening method to enlarge the coverage of regular screening population in the low-income and developing countries [18]. Our method is specially designed to instantly capture four characteristic spectral images that maximize the difference between normal and diseased tissues. Using our SNBI approach, four spectral images are captured with zero-time-lag in between and through a common optical path, hence they are spatially co-registered and can be readily fused into a combined image with enhanced contrast between normal and abnormal tissues. Our goal is to make the refresh rate of the contrast-enhanced fused image as high as a conventional white light colposcopy. With the fused image, a simple semi-automatic CAD method can be developed to classify the cervical tissue into different and clinical meaningful grades of tissue types. The critical objective of our method is to automatically detect lesions, grade them, and delineate their boundaries with minor supervision, and to do all of these at fast speed of a video-refresh rate. This is highly desirable for its future application, especially on a large clinical scale, in cervical cancer screening [1]. The advantages of the SNBI method include objective, invasive, chemical-free, instant results in vivo, and cost effectiveness. It is an ideal video camera to be integrated within a colposcopy to enlarge the coverage population of a cervical screening program in low-income countries and rural regions where medical resources are relatively scarce. The “Methods and Experiment” section 2 describes the SNBI method and validation experiment. The “Experimental Results” section presents the experimental results which indicate that the SNBI method in deeds enhance visual contrast and is capable to provide fast and objective diagnosis with weak supervision. The “Discussion” section discussed the advantages of the SNBI and its significance to the cervical cancer screening. A brief conclusion is disclosed in the “Conclusions” section.

## Methods and Experiment

### The SNBI Video Camera

**Fig. 1:**
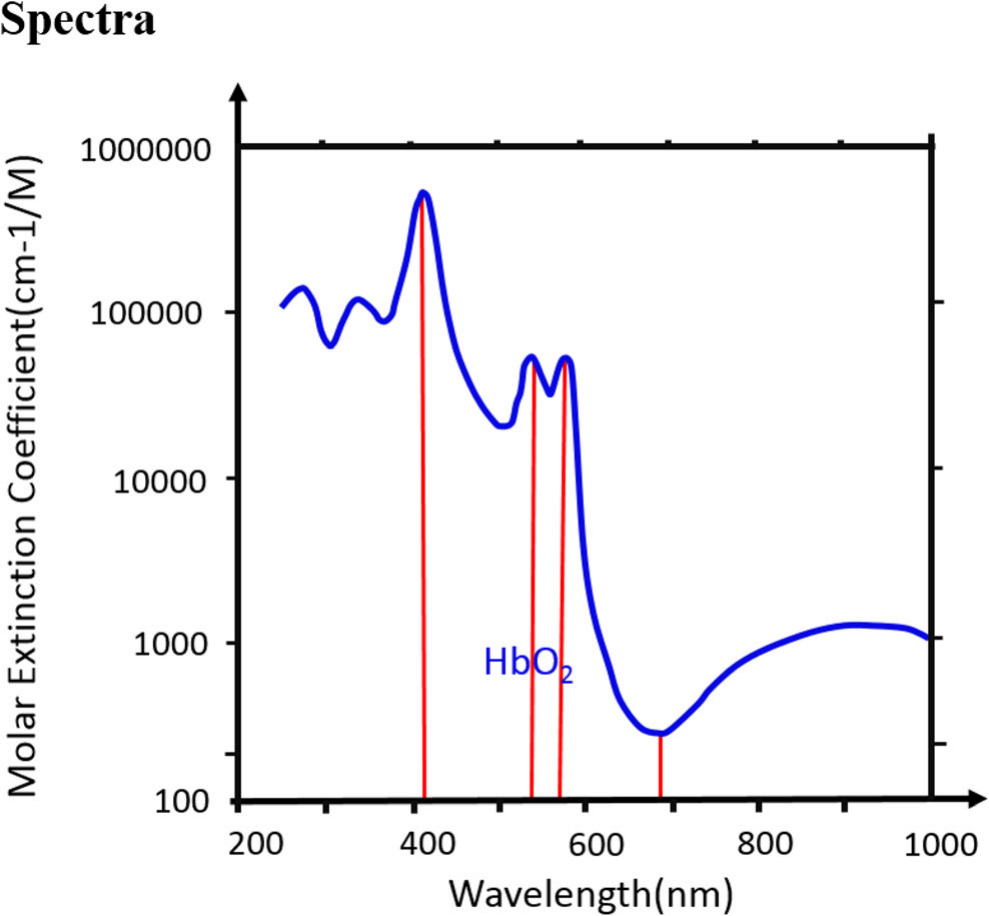
The optical extinction coefficient of hemoglobin

The image acquisition instrument of the SNBI method is a special narrow-band multi-spectral video camera, which is the integration of a monochrome imaging sensor with a miniatured narrow-band micro-arrayed spectral filter mosaic. This filter mosaic consists of four narrow passing bands periodically distribute in its two-dimensional surface grid, one spectral band at any spatial grid location. The center wavelengths of the passing bands of the spectral filter mosaic are determined according to the following reasons. It is evident from the literature that the cancerous tissue has vigorous metabolism and abundant blood flow. Compared with the surrounding normal tissues, the number of vessels, their blood flow speed, and the amount of hemoglobin are significantly higher in the cancerous region [3]. During the past decades, many efforts have been devoted to detect and measure the optical signature of hemoglobin in the form of a set of discrete wavelengths at which the chromophores have absorption or reflectance peaks [19]. Figure 1 shows the spectra of oxidized hemoglobin, which indicates the peaks absorption at 415 nm, 542 nm, and 577 nm, and peak reflectance at 525 nm and 696 nm. Marin and colleagues used the peak absorption of hemoglobin at 415 nm and valley absorption at 525 nm to classify cervical lesions into different grades [20]. Gustafsson and coworkers found that hemoglobin has significant absorbing peaks at wavelengths of 414, 542, and 577 nm (Fig. 1). Wang and colleagues proposed to use another three characteristic reflectance peaks of hemoglobin at wavelengths of 620, 696, and 772 nm to classify the cervical tissues into normal, inflammation, and high-grade lesions [7]. The characteristic bands used in this study are 415 ± 10, 450 ± 10, 525 ± 10, and 620 ± 10 nm. Among them, the 450 nm is not a characteristic band of lesion, but to serve as the background band.

**Fig. 2:**
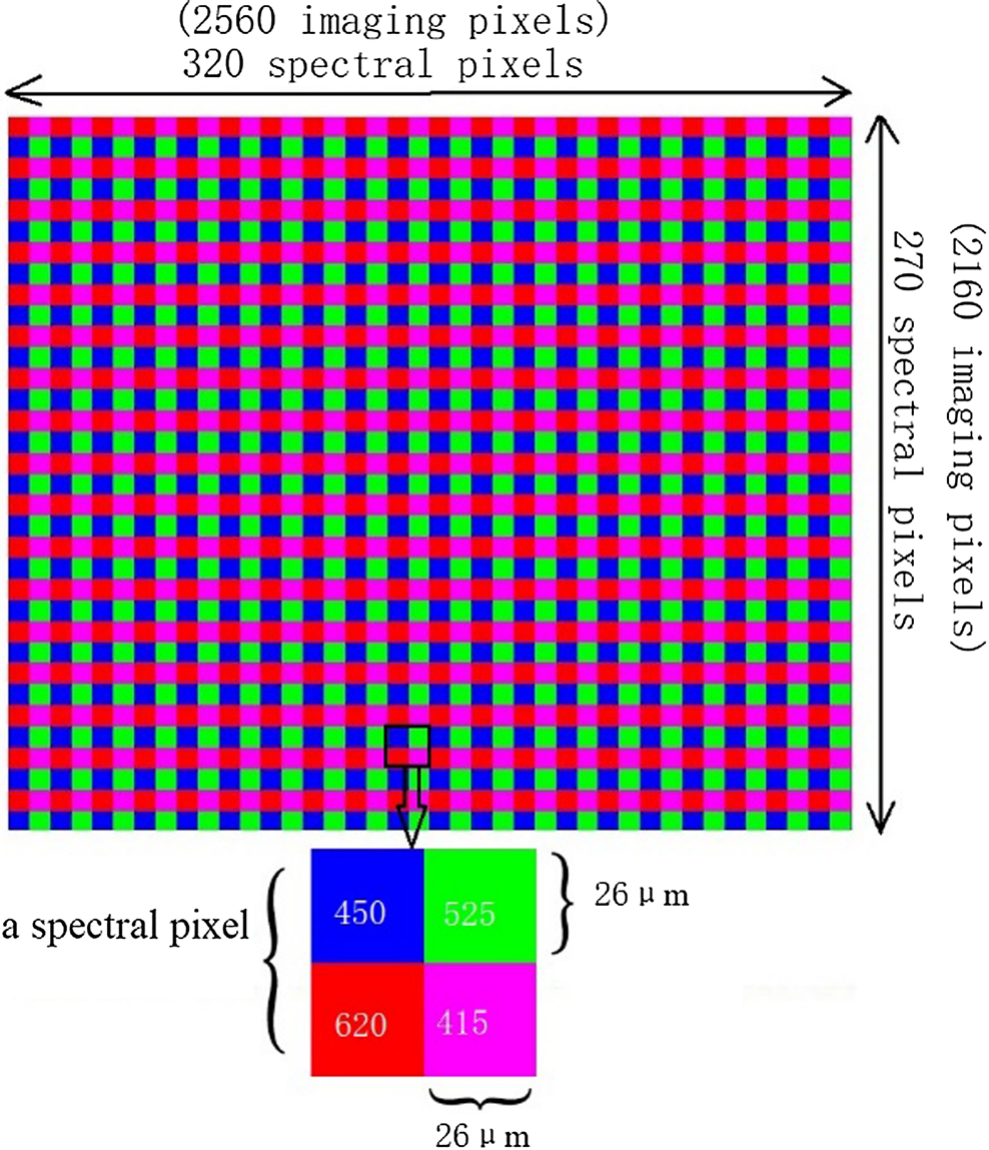
Illustration of the geometric arrangement of the micro-arrayed four narrow-band spectral filter mosaic, which is the core component of the SNBI video camera. It is of 16.6 mm in length, 14.0 mm in width, about 0.5 mm in thickness, and contains 320 × 270 spectral pixels. Each spectral pixel, in turn, contains four-squared sub-spectral pixels of the side length of 26 μm

**Fig. 3:**
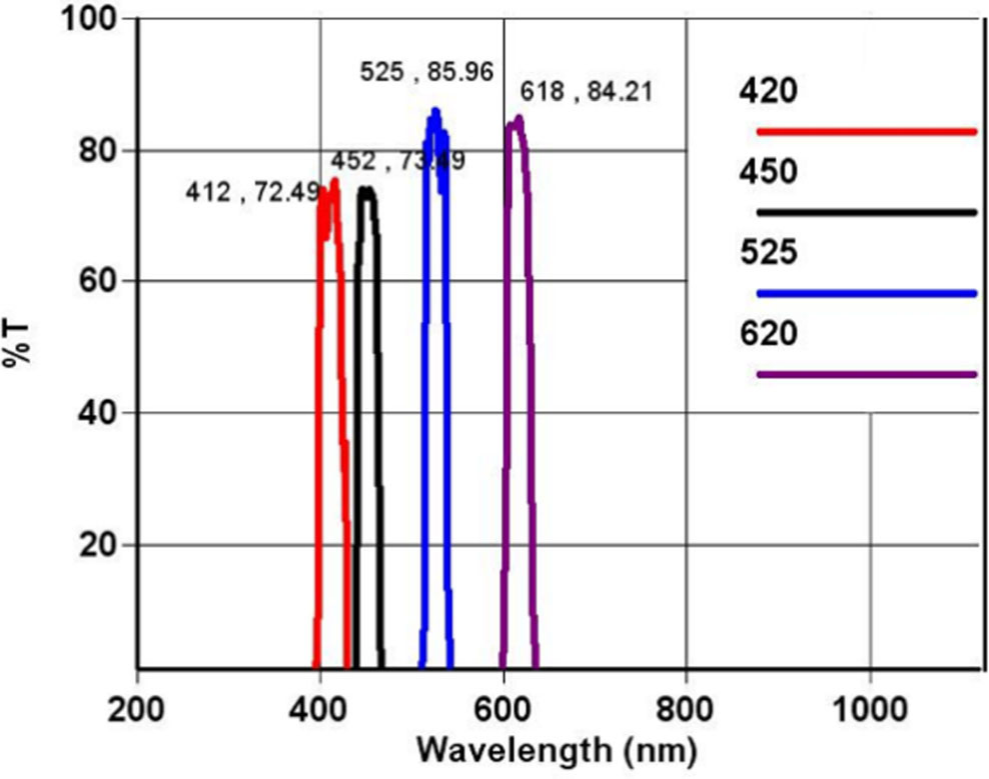
The measured transmittance curve of each spectral pixel containing four spectral sub-pixels corresponding to four narrow bands λ_1_ = 412, λ_2_ = 452, λ_3_ = 525, and λ_4_ = 970 nm. The minimum transmittance at passing bands is over 75%, and the maximum transmittance at stopping band is lower than 0.004%

The filter mosaic contains 320 × 270 spectral pixels (Fig. 2). Each spectral pixel is of square shape with a side length of 52 μm, containing four square–shaped spectral sub-pixel of side length 26 μm, allowing lights of one narrow spectral band to pass through while blocking light of other wavelengths [21, 22]. The transmittance rates at all four passing bands were over 70% and lower than 0.004% at all blocking bands, respectively (Fig. 3). Hence, the minimum optical density value (the logarithmic value of the ratio between the transmittance of the passing band and that of the blocking band) is higher than 4 [21]. The spatial gap between each spectral sub-pixel is, on an average of, 1 μm.

**Fig. 4:**
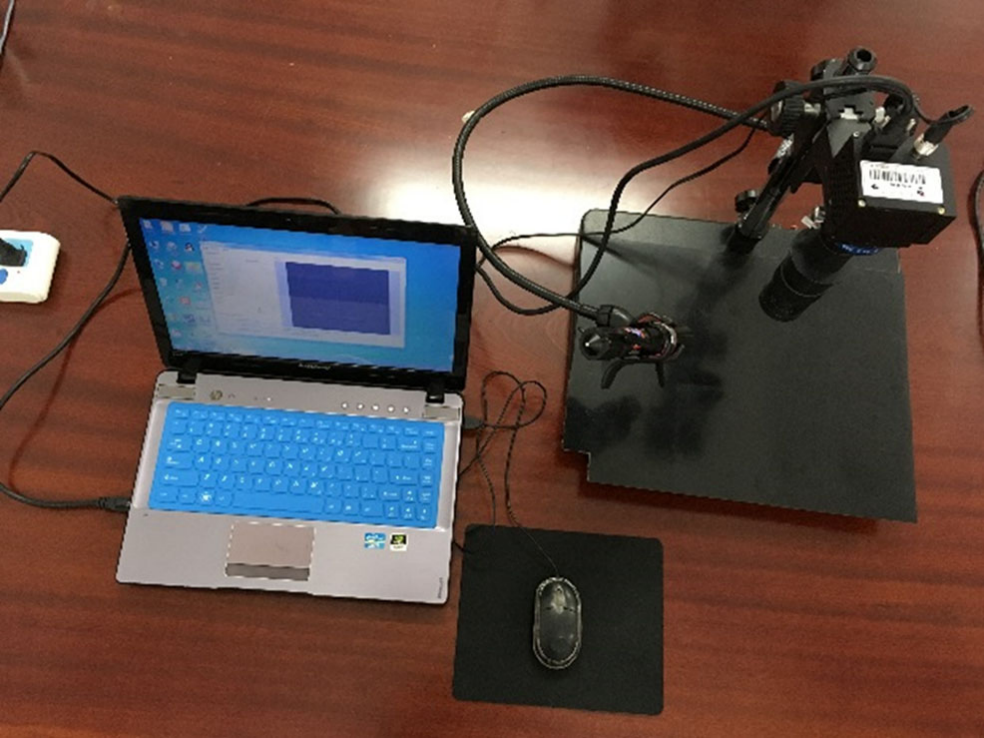
Experimental setup consisting of a miniature narrow-band snap-shot MSI system and a laptop. It was used for capturing MSI experimental data of freshly removed cervical cancerous sample tissues

The micro-arrayed four narrow-band spectral filter mosaic was integrated with an off-shelf monochrome SCMOS digital camera, resulting in a miniatured SNBI video camera (Ningbo 5-dimensional Inspection Science and Technology, at www.nb5d.com). The pitch number of the laminated off-shelf SCMOS imaging sensor was 6.5 μm, so each spectral pixel of the filter mosaic covered 8 × 8 imaging pixels of the imaging sensor, or one sub-spectral pixel covered 4 × 4 imaging pixels. The experimental setup used in this study consisted of a miniatured SNBI video camera and a laptop (Fig. 4).

### Snapshot Collection of Co-registered Multispectral Images of Cervical Tissues

**Fig. 5:**
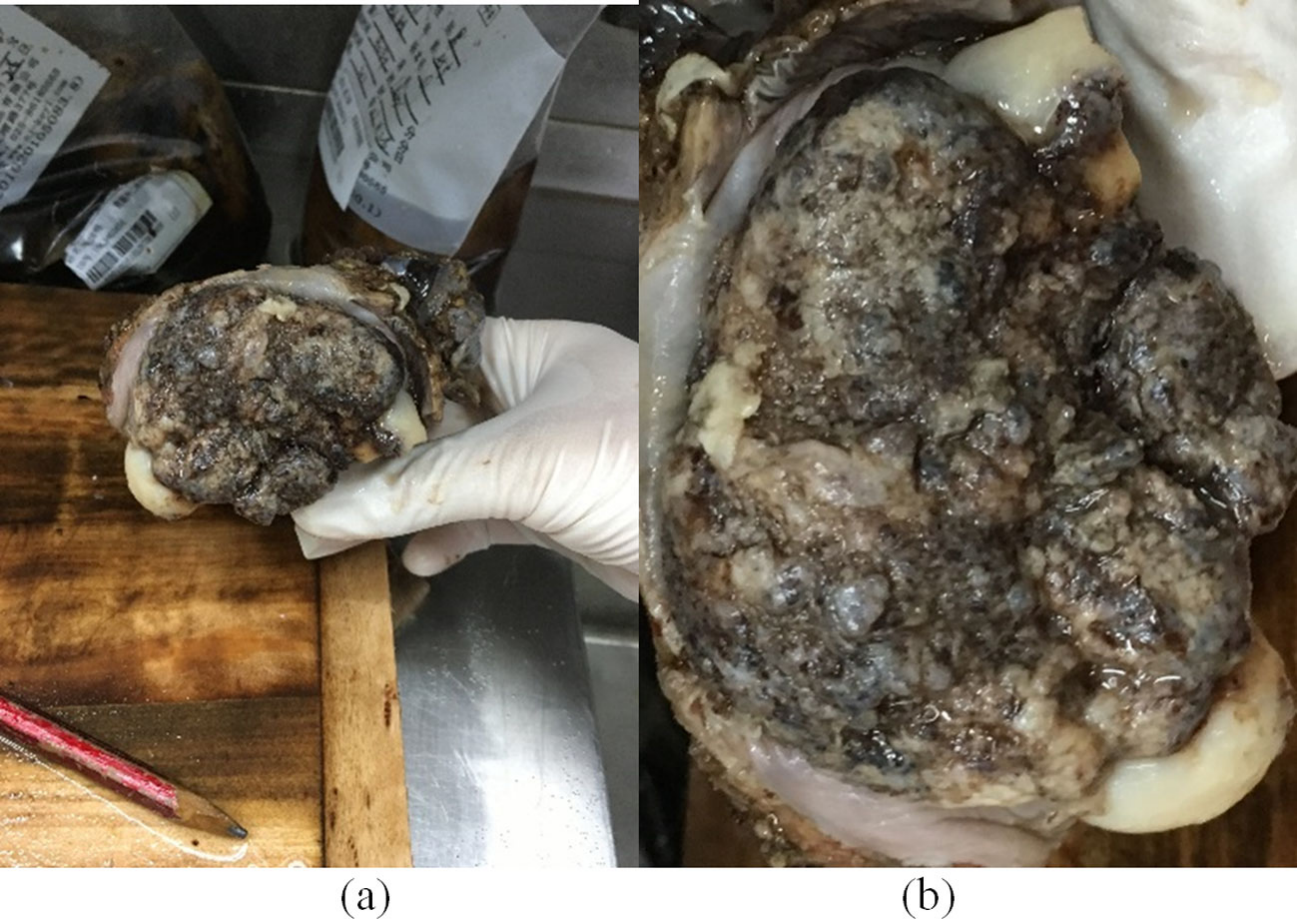
**a** Color image of one fresh cervical tissue sample, taken instantly after its removal from a patent. Its width is 52 mm, length of 58 mm. **b** Enlarged image of (**a**)

Currently, the miniature SNBI system was used to capture fresh cancerous tissues that are immediately removed by a surgical operation from a patient within 10 min (Fig. 5). This is acceptable because cancerous tissues that are removed by a conventional cone cutting surgical operation contain CIN3 or high-graded diseases also in general consist of other different types of tissues including normal, inflammation, CIN1, and CIN2. So far, cone cutting remained as a standard surgical treatment for cervical cancers, as there exists no precise surgical treatment for gynecology yet. Moreover, the reason why a typical cone cutting operation usually removes a much bigger area than the actual diseased region merely is for the safety concern, to ensure complete removal of the diseased tissue to reduce the occurrence rate.

**Fig. 6:**
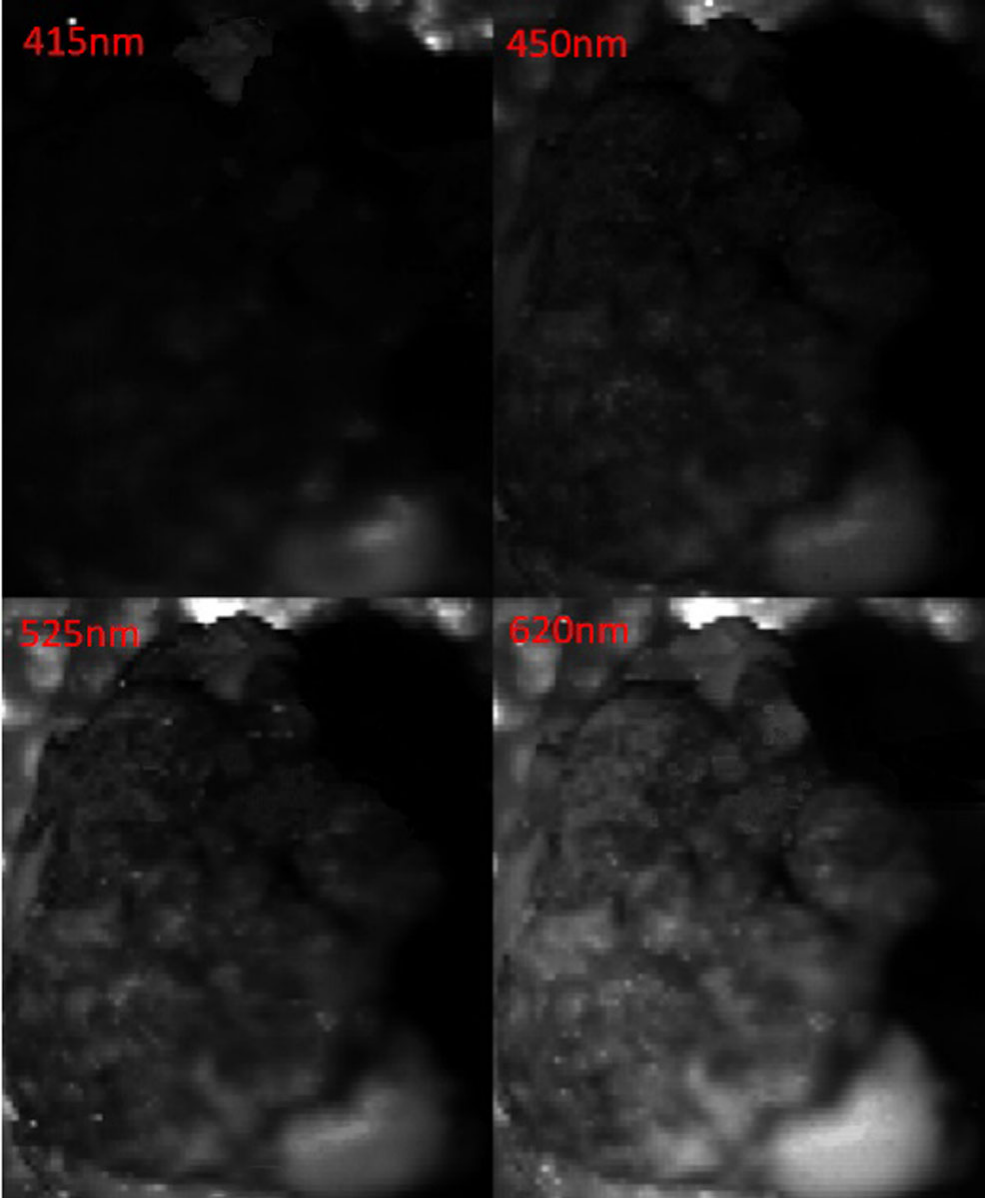
The simultaneously collected four characteristic narrow-band spectral images centered at 415 nm, 450 nm, 525 nm, and 620 nm of the sample, captured at a single exposure

The sample tissue was placed on the workbench below the miniature snapshot NBI system (Fig. 4). Under the illumination of a white light source which was available in a surgical operating room or in a typical office, the snapshot NBI system captured, with every single exposure, a single raw image of the cervical cancerous tissue. However, from everyone raw image, four spectral images centered at wavelengths of 415, 450, 525, and 620 nm were instantly split and displayed side-by-side (Fig. 6**).**

### Pre-processing

Before image analysis of the multispectral images, one pre-processing procedure was applied to normalize the four images to make them comparable by removing the effects of spectral-dependent illumination strength of the lighting source, the band-dependent quantum efficiency of the laminated SCMOS imaging sensor, and the uneven transmittance rate of the spectral pixels of micro-filter-arrayed mosaic. The equation used for this normalization procedure was:

1 $$ I\left(\lambda \right)=\frac{I{\left(\lambda \right)}_{\mathrm{sample}}-\overline{I{\left(\lambda \right)}_{dark}}}{\overline{I{\left(\lambda \right)}_{\mathrm{board}}}-\overline{I{\left(\lambda \right)}_{\mathrm{dark}}}} $$

where *I*(*λ*) is the normalized spectral image at a specific spectral band *λ* of the cervical tissue, *I*(*λ*)_sample_ is the corresponding original spectral image, and *I*(*λ*)_board_ and $\overline{I{\left(\lambda \right)}_{\mathrm{board}}}$ are the spectral image of a white Teflon board as a reference and its mean. $I{\left(\lambda \right)}_{\mathrm{dark}}\ \mathrm{and}\ \overline{I{\left(\lambda \right)}_{\mathrm{dark}}}$ are the spectral image of the dark current of the spectral system and its mean, respectively.

### Image Fusing

Although diseased cervical tissues have characteristic peaks at 425, 525, and 620 nm, any single spectral band alone was not enough to robustly differentiate diseased tissues from healthy ones, nor to classify diseased tissues into different grades. In this study, we fused the four narrow-band characteristic spectral images of cervical tissue into one to enlarge the difference between different diseased status. It was anticipated that the fused image might have an enhanced contrast between different tissue types than that of any single spectral image. The physiological idea behind the fusing algorithm was that the darker pixels in the spectral images *I*_415_ and *I*_620_ nm and whiter image of bands 525 nm were more likely related to cancerous tissues than normal region, as they contain more hemoglobin. The fusing algorithm used in this study was:

2 $$ {I}_{\mathrm{fused}}={\left(\frac{I_{620}-{I}_{450}+{I}_{415}-{I}_{525}}{a}\right)}^2 $$

**Fig. 7:**
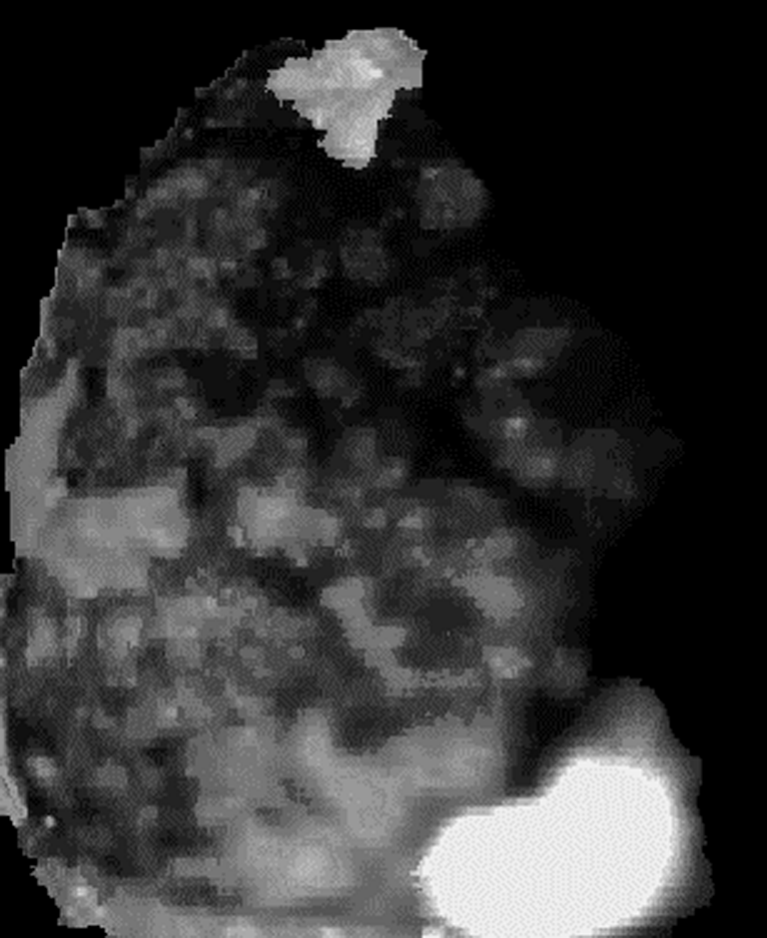
Exemplar fused image generated by the SNBI video camera

The constant *α* is a scale factor to ensure *I*_fused_ is within [0,255]. Using Eq. 2, through simple arithmetic operation containing addition and subtraction of the four narrow-band spectral images, one single fused image *I*_fused_ could be obtained*.* Every single exposure or frame of the MSI system corresponds to such a fused image. One fused image is shown in Fig. 7.

### Semi-automatic Classification of Cervical Tissues

**Fig. 8:**
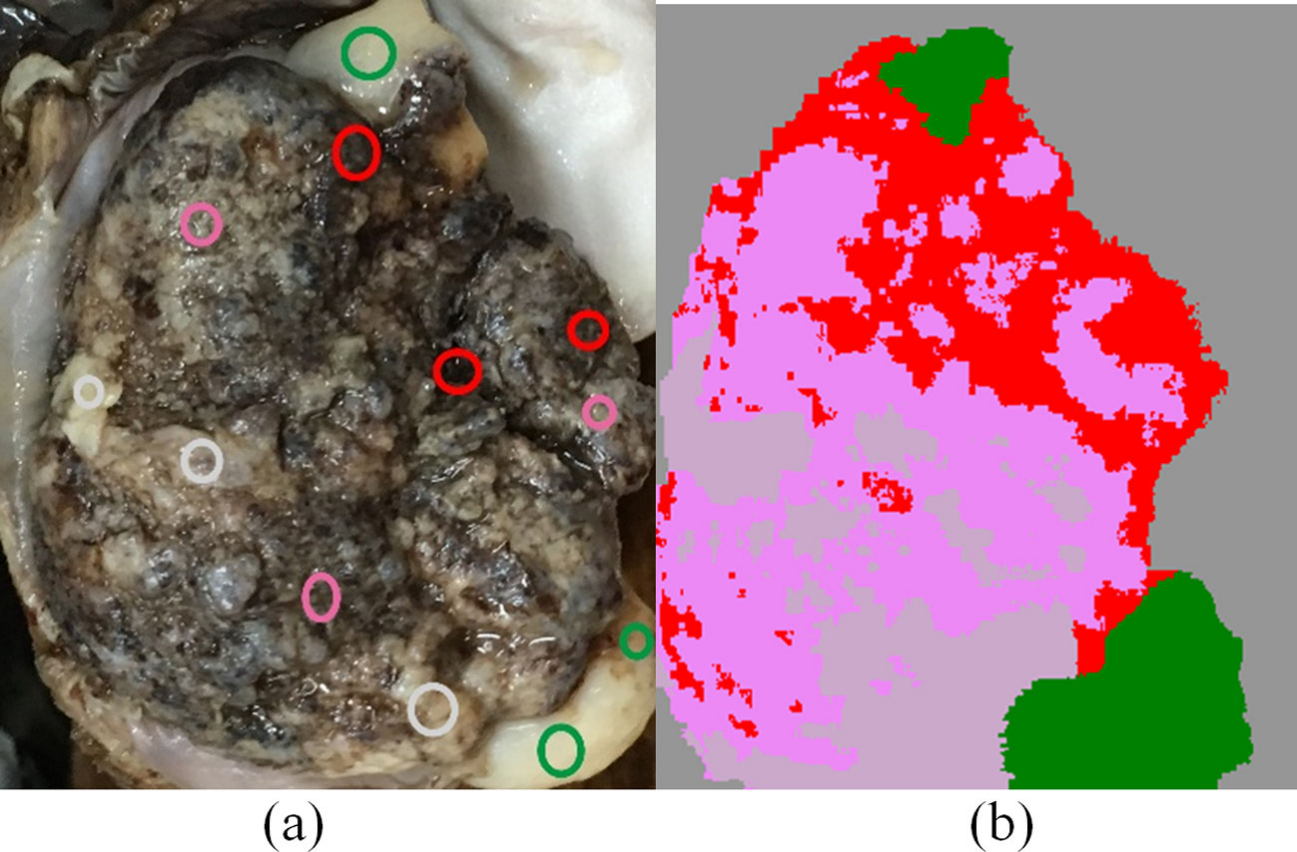
**a** Manual labels on a color image of cervical tissue, circles in green, gray, pink, and red indicate tissues of type normal, CIN2, CIN3, and carcinoma in situ, respectively. These labels are used as inputs to the computer-aided classification algorithm, **b** The classification result made by the Euclidean distance algorithm applied to the fused cervical tissue image generated by the proposed SNBI video camera, where green colored region indicates normal tissue, gray corresponds to CIN2, pink for CIN3, and red regions are carcinoma in situ

The fused image generally shows an apparent contrast between various types of tissues; hence, it is possible to use a simple classification algorithm to differentiate them. In this study, we used the Euclidean distance algorithm to classify CIN2, CIN3, and carcinoma in situ from normal tissues. The detailed procedure is described as follows. First, on a conventional color image of the cervical tissue, one or two regions of each type of tissue without given their boundary contours were manually labeled (Fig. 8a). Second, such manual labels were then roughly labeled on the fused image and were used as inputs for each type of tissue *S*^*x*^(where *x* is of the four possible types: normal, CIN2, CIN3, and carcinoma in situ). Then the distances of each remaining pixel on the fused image to all labeled pixels contained in every type of tissues *S*^*x*^ were calculated, and this pixel is classified as of type *x* that is corresponding to the smallest distance. At this moment, no strict registration was implemented between the color image and the fused spectral image. This is because the result is not so sensitive to the discrepancy of a couple of pixels of the labeled region. Besides, soon, this proposed method will employ two spatially registered cameras working together, one for conventional color image, the other for used spectral image.

The classification equation used is:

3a $$ {D}_{Ex}=\sqrt{\sum_{i=1}^p{\left(I-{S}_i^x\right)}^2} $$

3b $$ {C}_x=\min \Big({D}_{Ex\Big)} $$

where *I* is the intensity of the pixel to be classified, ${S}_i^x$ is the intensity of type *x* tissue of pixel *i* (*x* is one of the four types: normal, CIN2, CIN3, carcinoma in situ; *i* is a pixel of a total number of *p* pixels contained a particular labeled tissue type). *D*_*Ex*_ is the total distance between the pixel to be classified and all manual labels of type *x*. *C*_*x*_ is the smallest *D*_*Ex*_, and of that type *x* the pixel is classified into. The classification result is shown in Fig. 8b, where green area indicates normal tissue, gray corresponds to CIN2, pink as CIN3, and red as carcinoma in situ.

## Experimental Results

### Quantitative Comparison Between the SNBI Fused Spectral Image and Conventional WLI Colposcopy Image

**Fig. 9:**
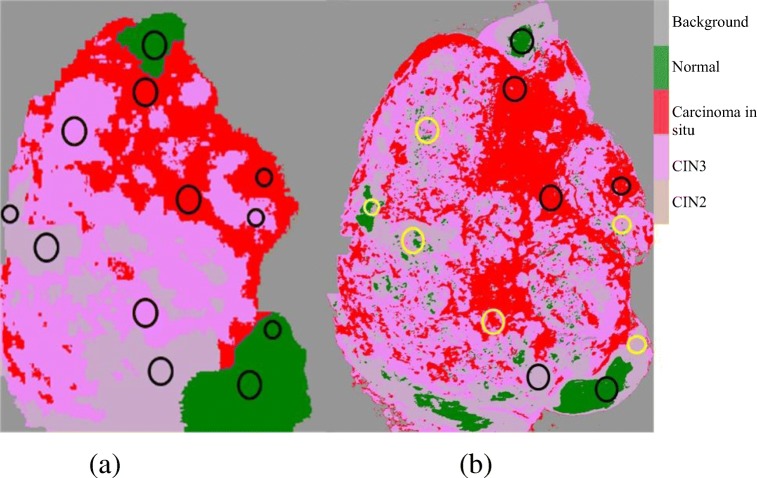
Comparison of classification result of same Euclidean minimum distance classification algorithm made on **a** the fused spectral image of this study and **b** the color image captured by a conventional colposcopy. Circles in black indicate classification agrees with the histopathological diagnosis, while circles in yellow indicate incorrect classification. Same sets of manual labels are used as inputs for both cases

An experiment was designed and conducted to answer two critical questions relevant to the proposed snapshot NBI method. First, whether or not the fused spectral image has indeed enhanced the contrast among the different types of tissues. Second, whether or not the contrast shown in the fused spectral image is high enough to differentiate different types of tissues. To answer the first question, the Euclidean distance classification method of Eq. (3) was applied to the color image captured by a conventional colposcopy (Fig. 8a) using manual labels shown in Fig. 8a as input. The classification results are as shown in Fig. 9b. For comparison, the classification made on the spectrally fused image is also shown in Fig. 9a.

The pathological diagnosis is used as the gold standard to evaluate and compare the accuracies of the same classification algorithm but made on two different images: the fused spectral image as well as the color image. As shown in Fig. 8 a and b, the black circles in both the images indicate the region where the CAD classification is consistent with the gold standard. In contrast, the yellow circles indicate that the CAD results are inconsistent with the histopathological diagnosis. The remaining regions are places where histopathological results are not available. Further as indicated by black circles that shown in Fig. 8a, at every location where the histopathological diagnosis is available, the CAD classification is consistent with the histopathological results. Table 1 is a summary of the accuracy comparison result of a Euclidean distance classification algorithm (EDCA) made on images captured by the proposed SNBI and a conventional color colposcopy, respectively. Based on this experiment, it is reasonable to conclude that the fused spectral image obtained by the miniature snapshot NBI method indeed enhanced the contrast between different types of tissues. These results reasonably suggest that the contrast shown in the fused spectral image between different tissue types is high enough to differentiate them automatically. This is in consistent with the finding made by Fakuma and coworkers who used a narrow-band imaging system to help diagnosing cervical carcinoma [7].

**Table 1:** Accuracy comparison of a Euclidean distance classification algorithm (EDCA) made on images captured by the proposed SNBI and a conventional color colposcopy, respectively

	Normal	Inflammation	CIN1	CIN2	CIN3	Carcinoma in situ	Accuracy
Number of samples	7	5	3	3	3	3	N/A
Proposed SNBI	7	5	3	3	3	3	100%
Conventional colposcopy	4	0	3	1	1	3	50%

### Subjective Evaluation by Multiple Clinics

**Fig. 10:**
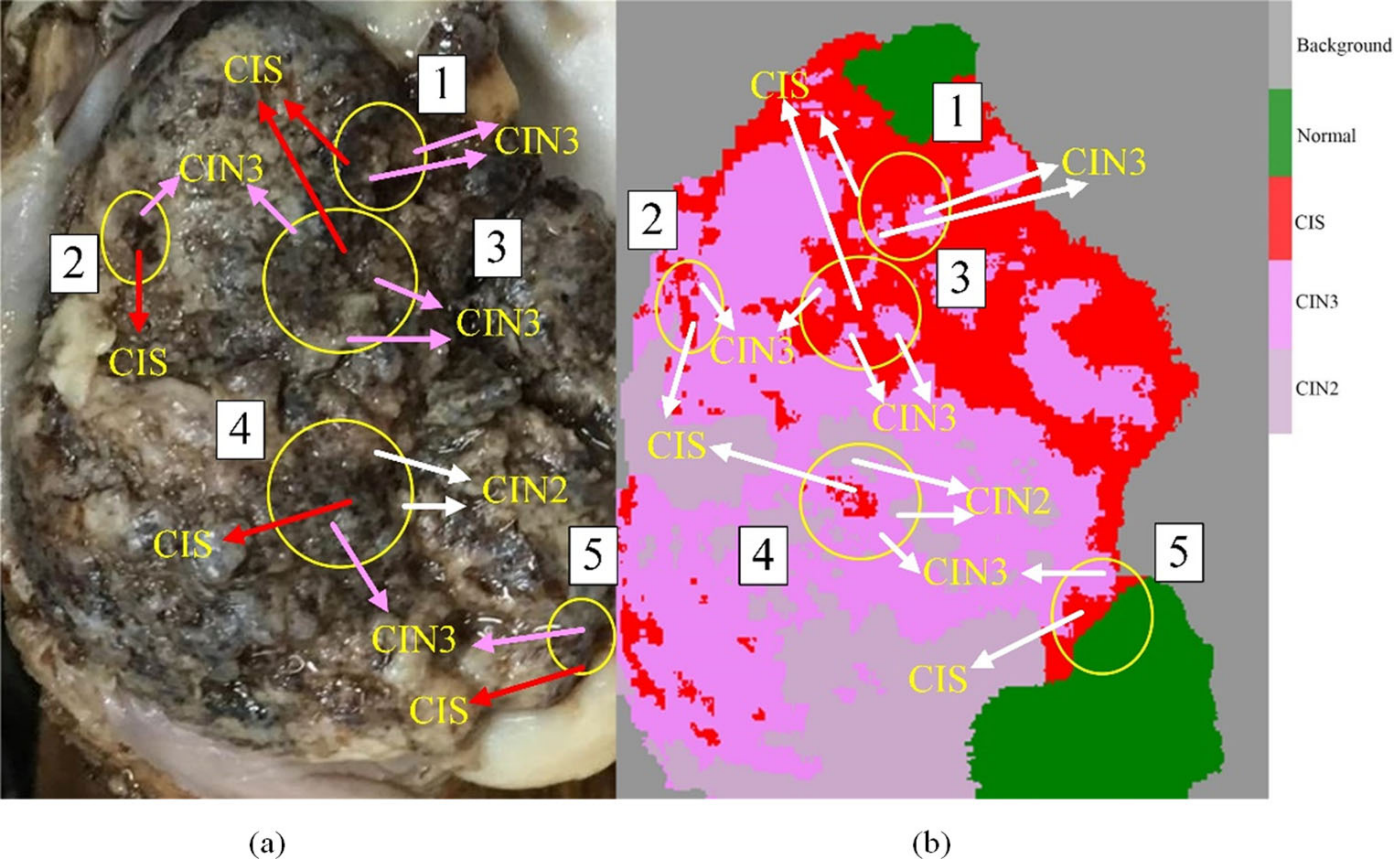
**a** The expert identified locations where multiple types of lesions co-exist in some tiny regions enclosed by yellow circles. **b** The yellow circles indicate the CAD correctly identified types of tissues, which are consistent with the experts’ consolation results

One of the samples used in this study is quite challenging as it contains two or more types of histologically diseased tissues in a few tiny regions. Several gynecologists admitted that it was difficult for them to decide which type of tissues in such a region should be classified into if based on the color image alone provided by a conventional colposcopy. After consulting with each other, they circled five such regions as indicated in Fig. 10a. For example, yellow circles labeled as 1, 2, 3, and 5 enclosing CIN3 and carcinoma in situ and circle 4 consisting of CIN2, CIN3, and carcinoma in situ. They found out that they could not agree with each other’s diagnosis made on conventional color image at zones 1, 3, and 4, as labeled in Fig. 10a. However, after careful evaluation of Fig. 10c, all five participating gynecologists were in consensus that they would agree with the Euclidean distance classification result shown in Fig. 10b. The result of this experiment is highly encouraging. First, the proposed method is helpful to provide a layout of quantitative results for multiple gynecologists to discuss upon. More importantly, it is capable of correctly classifying the challenging regions whether medical experts might find it is difficult to decide or may disagree with each other.

### Automatic Detection of Boundary Contours

**Fig. 11:**
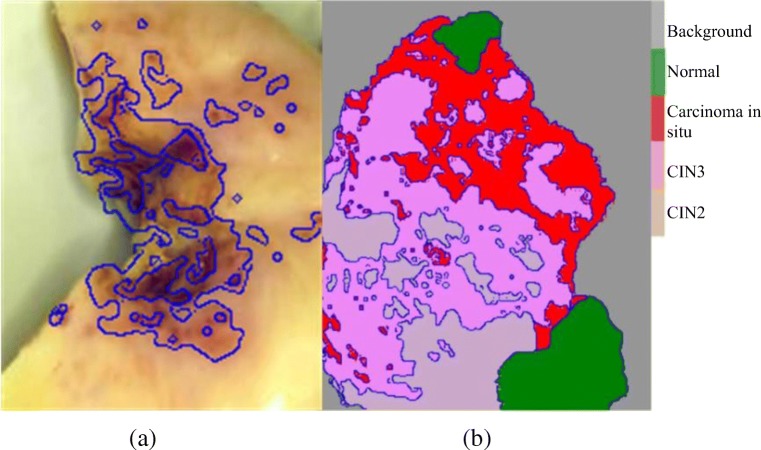
Two CAD classification results with boundary contours between different tissue types

Automatic detection of boundary contours of different tissue types is desirable for clinics, so they would know the location, size, and boundary of each type of diseased tissues. Therefore, special effort is devoted to automatically detect the boundary contours of different tissue types for the proposed SNBI method. A classical Canny edge detection algorithm is applied to the CAD classification results done on the fused spectral images of each cervical tissue samples, to enhance the boundary contours of each type of tissues. Figure 11 shows two exemplar resulting CAD classification results with enhanced boundary contours.

## Discussion

This paper proposed a snapshot NBI method with a semi-automatic CAD, i.e., a miniatured micro-arrayed SNBI method for automatic diagnosis. This SNBI method could instantly capture multiple images of cervix tissues at four characteristic bands centered at wavelengths of 415, 450, 525, and 620 nm. Since the four spectral images were spatially co-registered, they could be readily fused into a combined image with enhanced contrast between normal and abnormal tissues. With the fused image, a simple computer–aided diagnosis method is sufficient to classify the biological tissue into different and clinical meaningful grades of tissue types. This diagnosis method using Euclidean distance for classification, and it needed few doctor’s diagnoses as inputs. The preliminary results of the clinical evaluation experiment indicated that the method, without the aid of iodine and acetic acid reagents, could effectively classify the cervical tissue into meaningful pathological types of tissues. The classification is in good accordance with the common pathological diagnosis that acting as the current gold standard of clinical cervical cancer screening program.

The proposed snapshot NBI method with a semi-automatic CAD could achieve a highly efficient diagnosis at a refreshing rate equivalent to that of the underlying SCMOS monochrome camera, which is comparable to a conventional digital colposcopy. This is possible due to two reasons. First, the refresh rate of the generated fused spectral images is as high as the frame rate of the underlying SCMOS monochrome camera, as the multiple spectral images are captured with zero-time-lag-in-between at a single exposure. Second, only simple arithmetic operations are required to fuse multiple spectral images and the Euclidean distance classification algorithm contains only simple operations such as summation and comparison which can also be done in real time. The proposed SNBI method can update diagnosis result over 11 fps on a Pentium 1.6-GHz laptop. It is reasonable to conclude that the goal of achieving real-time video rate diagnosis by the proposed snapshot NBI method is proved to be feasible.

One interesting feature of the proposed SNBI method is its capability to accurately classify challenging regions where different medical experts may find it is hard to accept each other’s opinion. Further, the graphical layout of the quantitative classification results provides a convenient communication channel for the medical experts to discuss upon. Another advantage of the proposed SNBI method is its capability of detecting the boundary contours of different tissue types (Fig. 10). This is desirable for the clinics to know the exact location, size, and boundary of the diseased region, especially during image-guided sample collection procedure and precise surgical treatment.

Currently, we are integrating the SNBI video camera with an auto-focusing optical system with adjustable optical zoom from × 2 to × 26. After this, we may conduct an in vivo preclinical study using the SNBI imaging system to formally validate its diagnostic value for cervical cancer screening.

## Conclusions

The proposed snapshot narrow-band imaging (SNBI) method enhances contrast and improves computer classification accuracy over conventional white light imaging (WLI) methods. The boundary contour between health tissue, cervical precancerous regions, and carcinoma in situ can be automatically delineated in SNBI. The SNBI contrast–enhanced method could make objective diagnostic result instantly and invasively with weak supervision where the expert identifies the presence of only a couple of lesions without explicating their boundaries. It generates automatic diagnostic results with clear boundary contours at over 11 fps on a Pentium 1.6-GHz laptop.

Further, it is miniature in size, cost-effective, and convenient to operate without the need of a sequence of data acquisition operations. Hence, the proposed SNBI is of great significance to enlarge worldwide the coverage of regular cervical screening program especially in low-income countries, and to live guide surgeries such as biopsy sample collection and accurate cervical cancer treatment. Although validated only with cervical cancerous tissues, in principle SNBI should work with any hemoglobin rich/scarce lesion detections.
